# A Data-Driven Method for Determining the Loading Spectrum in Fatigue Tests for Working Device of Engineering Machinery

**DOI:** 10.3390/s26154724

**Published:** 2026-07-25

**Authors:** Yuqi Li, Wudi Yue, Xiaoming Wang, Yaozhi Xiong, Bing Li

**Affiliations:** 1 School of Mechanical and Automotive Engineering, Guangxi University of Science and Technology, Liuzhou 545006, China; 20250101002@stdmail.gxust.edu.cn (W.Y.); 100001548@gxust.edu.cn (B.L.); 2 Guangxi Earthmoving Machinery Collaborative Innovation Center, Guangxi University of Science and Technology, Liuzhou 545006, China; 3 Guangxi Liugong Group Company, Ltd., Liuzhou 545007, China; wangxm@liugong.com (X.W.); xiongyz@liugong.com (Y.X.)

**Keywords:** fatigue test, NARX model, nonlinear system identification, inverse virtual iteration

## Abstract

**Highlights:**

**What are the main findings?**

- A data-driven modeling method based on a multi-input single-output NARX model is proposed, which uses measured input–output data to accurately characterize the nonlinear dynamic behavior of a loader’s working mechanism.
- A genetic algorithm-based inverse virtual iteration technique is designed to invert the excitation load spectrum at the bucket edge—which cannot be measured directly—by targeting measurable structural responses (output loads).

**What are the implications of the main findings?**

- This effectively solves a key engineering challenge: because certain position sensors cannot be installed on the machine, it is impossible to obtain the input load.
- It provides a low-cost, high-efficiency solution for generating fatigue test load spectra on a test bench, enabling the structural fatigue design of working mechanisms without the need for direct testing, thereby significantly shortening the R&D iteration cycle.

**Abstract:**

The working device of a wheel loader is a critical component of construction machinery and is subjected to long-term random loads during operation, making it prone to fatigue damage. To evaluate whether it satisfies the design life requirements, load spectra are typically extracted and fatigue life analysis is performed. However, the determination of the load spectrum remains a key challenge, as no standardized method is currently available in the field of construction machinery. To this end, this paper proposes an inverse VIM based on a data-driven model and a genetic algorithm to invert the input load spectrum and apply it to the working device of a loader, so as to realize the fatigue life assessment of the working device of a construction machinery in order to guide its structural design. The initial step involves applying random loads to the four actuators of the fatigue testing platform and measuring the corresponding output loads at various measurement points. By utilizing data identification techniques and the NARX model, a numerical model for the working device of the loader is established. Subsequently, employing genetic algorithms and the measured target output loads, the input loads are inversely determined. Finally, the obtained input loads are applied to the fatigue testing platform, and the output loads at different measurement points are collected and compared with the target output loads to validate the effectiveness and reliability of this method in terms of damage assessment. The results indicate that the inverse virtual iteration technique based on the NARX model and genetic algorithm provides a useful reference for determining the loading spectrum of construction machinery.

## 1. Introduction

As a critical part of the equipment industry, engineering machinery underpins infrastructure construction, and the fatigue life of its key components is a vital indicator of safety and reliability. Fatigue testing can simulate the cyclic loads that structures experience in practical applications and evaluate their durability by observing the accumulated damage caused by these cyclic loads. However, this testing process is time-consuming, expensive, and subject to environmental limitations. To overcome these limitations, engineers often employ bench fatigue testing, which accelerates cumulative damage by increasing load amplitude, frequency, or temperature. A prerequisite for bench fatigue testing is that the applied load must accurately reflect the impact of real loads on structural fatigue life. However, in practical measurements of the loads acting on the working device (e.g., the bucket and linkage mechanism) of a wheel loader, it is often challenging to directly obtain the input load. Therefore, determining the load spectrum remains a key challenge that must be addressed.

In the automotive industry, a common approach involves placing sensors on critical components such as wheels, engine, and chassis to collect vehicle operating signals on endurance roads. The measured signals are subsequently preprocessed through filtering and outlier removal, and then converted into a program load spectrum suitable for bench testing. This enables fatigue life analysis and prediction for automotive components. Kim et al. [1] combined a four-post road simulator based on the Virtual Iteration Method (VIM) with axle acceleration inputs, and used the VIM to obtain accurate design loads. They determined the stress distribution in the tractor frame under unit excitation. Zhou et al. [2] developed a full vehicle multi-body dynamics model based on measured vehicle parameters. Using the VIM, they obtained the equivalent excitation of the load spectrum obtained from road spectrum acquisition tests and applied it to the Multi-Body Dynamics (MBD) model, resulting in the load spectrum at critical points of the rear axle. Li [3] proposed a hybrid physical prediction method that combines finite element analysis with aerospace material test data to efficiently and accurately predict low-cycle fatigue life under various multi-axial loading conditions. Yu et al. [4] proposed a load spectrum editing technique aimed at accelerating test speeds and reducing testing durations.

In the previous study, the use of the VIM necessitates the establishment of a numerical model between inputs and outputs. Based on this model, virtual iterations are performed using measured output signals to deduce the input signals. In the automotive industry, the input loads on wheels are back calculated using response data obtained from a four-poster test bench. However, this mature industrial workflow is only suitable for vehicle structures and cannot be directly applied to construction machinery. Unlike the automotive industry, the harsh operating environment of loaders and the significant variations in random loads under different operating conditions make it difficult to install sensors directly on the bucket cutting edge to measure the external loads acting on the working equipment. Furthermore, fatigue bench testing of the working device in construction machinery is typically conducted under fixed configurations, which is inconsistent with the time-varying operating conditions encountered in actual operation. Currently, there are not commercially available MBD software tools or industry standards specifically designed for this type of heavy machinery to address the problem of load spectrum inversion. Consequently, we propose a method for determining the fatigue load spectrum of the working device in engineering machinery, effectively replacing the conventional—and often inaccessible—MBD-based inverse iteration framework specific to the automotive sector. This would enable estimation of the fatigue life of the working device and guide its structural design.

Currently, three common modeling methods are used: the Finite Element Method (FEM), mechanical modeling, and data-driven modeling. The finite element method can provide fairly accurate results through mesh refinement. Xu et al. [5] proposed a calibrated finite element method-discrete element method model to investigate the impact of the interaction between off-road tires and granular terrain on the traction performance of off-road vehicles. The model was validated through stiffness tests and triaxial compression tests. Kumar et al. [6] proposed a parametric finite element modeling method for lumbar pedicle screws, with the aim of investigating the effects of friction forces between all contact surfaces. However, FEM is time-consuming and relies heavily on boundary conditions and element quality. Mechanical modeling involves abstracting and generalizing basic mechanical models from real-world engineering and then transforming them into mathematical models for solution. Yin et al. [7] developed a hydraulic model incorporating both sedimentation and mechanical loading to examine the effects of internal erosion in engineering structures. The reliability and applicability of the model were demonstrated through experimental testing and a case study involving dam foundations. Antonio et al. [8] proposed a mesoscale mechanical model that enhances the shear strength of concrete slabs; this model is used to study the mechanical behavior of concrete slabs under concentrated loads. The validity of this mesoscale mechanical model was verified through experimental testing of the mechanical properties of steel fiber-reinforced concrete. However, mechanical modeling may not fully consider all aspects of the actual system, leading to discrepancies between the model and reality. Data-driven modeling exhibits strong non-linear fitting capabilities, combining time series data with external input data. It can make predictions in non-stationary environments, handle complex non-linear relationships, and is less influenced by experiential factors. Compared to the other two methods, data-driven modeling is simpler, faster, more versatile, and has numerous engineering applications. Yang et al. [9] proposed a model framework based on empirical and data-driven methods to investigate potential associations between genes and diseases. They also developed a multi-orthogonal search algorithm applicable to various operating conditions and validated the applicability of the model using experimental data. He et al. [10] tackled the issue of real-time estimation of petroleum flow in pipelines, considering the influence of noise and instrument accuracy that hinder direct observation. They proposed an approach that combines data-driven and model-driven techniques to estimate real-time petroleum flow and improved the prediction strategy for local pressure transients. The research results demonstrated the enhanced accuracy of petroleum flow estimation in unforeseen circumstances.

Since its introduction by Billings et al. [11,12] in 1985, Nonlinear Auto-Regressive with exogenous (NARX) model has been widely adopted for nonlinear system identification due to its simplicity and broad applicability. As a data-driven numerical model, it is characterized by its simplicity, fast modeling speed, and wide applicability to a broad range of nonlinear systems, making it widely adopted. With the advancement of technology, several classical modeling algorithms have been introduced, such as the Orthogonal Least Squares (OLS) algorithm [13,14] and the Forward Regression Orthogonal Least Squares (FROLS) algorithm [15]. By treating grey-box identification as a multi-objective problem, the method balances biases in the modeling process and obtains a numerical model that represents the dynamic and static behavior of the system. Kim et al. [16] combined the NARX model with self-organizing map to establish flood prediction models for each basin in Seoul. They conducted flood predictions based on the data from major rainstorms in Seoul in 2010 and 2011, and the results showed high accuracy in flood forecasting. Wu et al. [17] aimed to predict behavioral risk features of drivers in tunnels. They conducted real-world experiments in two long-distance tunnels, collecting vehicle speeds from both experienced and inexperienced drivers. Using the NARX model, they developed a driver behavior risk quantification model based on safety speed differentials. The above studies indicate that combining NARX with evolutionary algorithms holds great potential in the field of system identification and control. This enables us to apply this method to address inverse load identification problems in heavy machinery.

This paper proposes a data-driven method for inferring input load spectra and applies it to fatigue analysis of a wheel loader working device. To obtain the excitatory loads of the working device, a data-driven model is established for the mechanical model of the large-scale engineering machinery working device. The data-driven model, combined with a genetic algorithm, is used to infer the fatigue loading spectrum based on measured data. Finally, the identified loads are applied to the fatigue test bench to validate the method’s effectiveness and feasibility. The remainder of this paper is organized as follows: Section 2 states the problem, highlighting the difficulties in load spectrum acquisition; Section 3 details the NARX modeling methodology, the FROLS parameter identification algorithm, and the configuration of the genetic algorithm; Section 4 presents a numerical example using a two-degree-of-freedom system to verify the theoretical framework; Section 5 describes the experimental application on a wheel loader test rig and analyzes the results; and Section 6 summarizes the main conclusions.

## 2. Problem Statement

**Figure 1:**
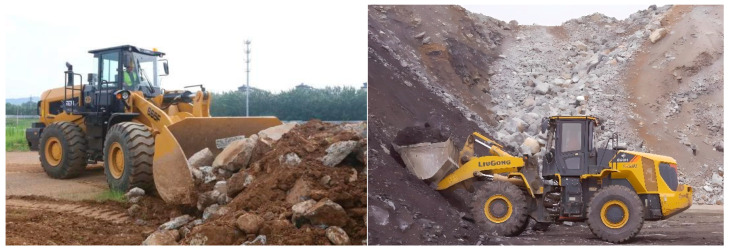
The different operating conditions of a wheel loader.

Figure 1 illustrates a wheel loader operating under harsh and variable conditions, where its working mechanism (as the primary actuator) is subjected to fluctuating loads over extended periods, making it highly susceptible to fatigue failure. Moreover, fatigue failure exhibits strong unpredictability and concealment, often occurring without apparent signs, posing potential threats to personnel and equipment safety. Therefore, the fatigue life reliability of the wheel loader’s working mechanism is a critical indicator of the overall machine quality. Accurately assessing the reliability of the working device plays a vital theoretical and practical role in guiding the design, production, and usage of wheel loaders, thereby improving their performance and lifespan. Currently, two primary methods are commonly used for fatigue testing of the wheel loader working device. The first method involves hiring operators to perform repetitive tasks until fatigue failure occurs in the working device. However, due to variations in operator technique and the influence of weather conditions, this method has poor reproducibility and is both costly and time-consuming. The second method involves designing a dedicated fatigue test bench for the wheel loader working device. By increasing the load amplitude or frequency, the speed of damage accumulation is enhanced, thus shortening the testing time and effectively overcoming the limitations of the previous method. To ensure the validity of bench fatigue testing, the input load should accurately reflect real operating loads affecting the fatigue life of the working device. Specifically, it should replicate the random load experienced by the cutting edge of the loader bucket during operation. Currently, there is no publicly available standard for collecting input loads, and it is difficult to directly measure the random loads at the cutting edge by placing sensors. To address this, a data-driven model combined with a genetic algorithm is proposed to iteratively determine the input load spectrum based on the measured output load spectrum, allowing for the reproduction of strains experienced during operations.

A data-driven model can utilize a large amount of data for training and learning, thereby enhancing its accuracy by fully leveraging the available information. The model can better capture patterns and trends within the data, leading to more precise predictions. Additionally, data-driven models have the capability to automatically learn from and adapt to new data. Once new data becomes available, the model can update itself through retraining to better accommodate new situations and changes. This flexibility enables data-driven models to handle continuously evolving problems effectively. Furthermore, data-driven models offer interpretability by allowing analysts to analyze the model’s internal structure and parameters, gaining insights into how the model makes predictions. This interpretability aids in understanding the model working principles, enhances trust in model performance, and facilitates evaluation of the reasonableness of model outputs. By applying data-driven models, a numerical model can be established for the working device of a loader to represent its system model. With both the numerical model of the working device and the measured output load spectrum, it becomes possible to use genetic algorithms to inversely determine the input load spectrum.

## 3. Dynamic Modeling Method Based on the NARX Model

### 3.1. Description of the NARX Model

The traditional NARX model can be implicitly defined as follows [18,19,20]:

(1) $$\begin{array}{l} y(t)=F\left[y(t-1),y(t-2),\cdots ,y\left(t-n_{y}\right),\right.u(t-d)\\ \;\;\;\;\left.u(t-d-1),\cdots ,u\left(t-d-n_{u}\right)\right]+e(k) \end{array}$$

where *e*(*k*) represents an independent signal relative to the input–output signals; *d* is a time delay; *n_u_* and *n_y_* are the maximum time delays for the input and output sequences, respectively.

When the time delay *d* = 1, the discrete-time single-input single-output NARX model can be formulated as follows:

(2) $$\begin{array}{l} y(t)=F\left[y(t-1),\cdots ,y\left(t-n_{y}\right),u(t-1),\cdot \cdot \cdot ,u\left(t-n_{u}\right)\right]\\ \;\;\;=\theta_{0}+\sum_{i_{i}=1}^{n}\theta_{i_{1}}x_{i_{1}}(t)+\sum_{i_{1}=1}^{n}\sum_{i_{2}=i_{1}}^{n}\theta_{i_{1}i_{2}}x_{i_{1}}(t)x_{i_{2}}(t)+\cdots +\sum_{i_{1}=1}^{n}\\ \;\;\;\;\cdots \sum_{i_{l}=i_{l-1}}^{n}\theta_{i_{1}i_{2}\ldots i_{l}}x_{i_{1}}(t)x_{i_{2}}(t)\cdots x_{i_{l}}(t)+e(k) \end{array}$$

where *F*[.] is the unknown function to be identified; *u*(*t*) and *y*(*t*) represent the input and output signals, respectively; *n_u_* and *n_y_* are the time delays of *u*(*t*) and *y*(*t*), respectively; *l* is the degree of the model (2); *t* represents the *tth* data point and without any unit; *θ_i_* are coefficients; *n* = *n_u_* + *n_y_*; and

(3) $$x_{i}(t)=\left\{\begin{array}{c} y(t-i)\\ u(t-i+n_{y}) \end{array}\right.\;\;\begin{array}{c} 1\leq i\leq n_{y}\\ n_{y}+1\leq i\leq n=n_{y}+n_{u} \end{array}$$

where *t* represents the *tth* data point in the data set.

Equation (2) is a deterministic model describing a wide range of nonlinear systems, whose parameters can be identified using time-domain input–output data. It can also be expressed in a linear-in-parameter regression form as follows:

(4) $$y(t)=\sum_{i=1}^{M}\theta_{i}p_{i}(t)+e(k)$$

where *p_i_*(*t*) represents the model terms, *θ_i_* is a coefficient, and *M* is the number of total candidate model terms. The number of candidate model terms can be computed as follows:

(5) $$M=\frac{(n+l)!}{n!l!}$$

where *n* is the sum of the maximum time lag of the input and output regressors, and *l* is the degree of model (2).

Equation (4) can thus be represented in a compact matrix form as

(6) y=Pθ+e

where *y* represents the system output matrix; *P* is the regression matrix formed by the candidate model terms, $p=[p_{1}(t),p_{2}(t),\ldots ,p_{M}(t)]$; and *θ* is the coefficient matrix, $\theta =[\theta_{1},\theta_{2},\ldots ,\theta_{M}]$.

### 3.2. Multi-Input Single-Output NARX Model Architecture

The traditional NARX models mainly focus on single-input single-output scenarios, which fail to fully capture the complexity and diversity of the system. Therefore, a more flexible and comprehensive approach is required, known as the multi-input single-output NARX model. This method better adapts to real-world variations, enhancing the accuracy and predictive capabilities of the model.

The multi-input single-output NARX model can be implicitly defined as follows:

(7) $$\begin{array}{l} y(t)=F[y(t-1),y(t-2),\ldots ,y(t-n_{y}),u_{1}(t-d),u_{1}(t-d-1),\ldots ,\\ \;\;\;\;u_{1}(t-d-n_{u}),\ldots ,u_{m}(t-d),u_{m}(t-d-1),\ldots ,u_{m}(t-d-n_{u})]+e(k) \end{array}$$

where *e*(*k*) represents an independent signal relative to the input–output signals; *d* is a time delay; *u*(*t*) and *y*(*t*) represent the input and output signals, respectively; *n_u_* and *n_y_* are the maximum time delays for the input and output sequences, respectively.

When the time delay *d* = 1, the discrete-time multiple-input single-output NARX model can be formulated as follows:

(8) $$\begin{array}{l} y(t)=F[y(t-1),\ldots ,y(t-n_{y}),u_{1}(t-1),\ldots u_{1}(t-n_{u}),\ldots ,u_{m}(t-1),\ldots u_{m}(t-n_{u})]\\ \;\;\;\;\;\;\;=\theta_{0}+\sum_{i_{i}=1}^{n}\theta_{i_{1}}x_{i_{1}}(t)+\sum_{i_{1}=1}^{n}\sum_{i_{2}=i_{1}}^{n}\theta_{i_{1}i_{2}}x_{i_{1}}(t)x_{i_{2}}(t)+\cdots +\sum_{i_{1}=1}^{n}\cdots \sum_{i_{l}=i_{l}-1}^{n}\theta_{i_{1}i_{2}\cdots i_{l}}x_{i_{1}}(t)x_{i_{2}}(t)\cdots x_{i_{l}}(t)+e(k) \end{array}$$

where *t* represents the *tth* data point in the data set, *l* is the highest nonlinearity order of model (8), *θ_i_* represents the corresponding coefficients of each *x_i_*(*t*) term, *n* = *n_u_* + *n_y_*, where *x_i_*(*t*) is:

(9) $$x_{i}(t)=\left\{\begin{array}{c} y(t-i)\\ u_{m}(t-i+n_{y}) \end{array}\right.\;\;\begin{array}{c} 1\leq i\leq n_{y}\\ n_{y}+1\leq i\leq n_{u}+n_{y} \end{array}$$

Equation (8) can also be described in the following linear regression form:

(10) $$y(t)=\sum_{i=1}^{M}\theta_{i}p_{i}(t)+e(k)$$

where $p_{i}(t)=p_{i}(x(t)),\;i=1,\ldots ,M$ represents the model terms; *e*(*k*) represents an independent signal relative to the input–output signals; *θ_i_* is a coefficient, and M is the number of total candidate model terms. The number of candidate model terms can be computed as follows:

(11) $$M=\frac{(n+l)!}{n!l!}$$

where *n* = *n_u_* + *n_y_*, and *l* is the highest nonlinearity order of model (8).

Equation (10) can be further expressed in the following matrix form:

(12) y=Pθ+e

where $y={\left[y(1),\ldots ,y(N)\right]}^{T}$ represents the system output vectors; N represents the number of sampling points; and θ is the coefficient matrix, $\theta ={\left[\theta_{1},\theta_{2},\ldots ,\theta_{M}\right]}^{T}$; P is the regression matrix formed by the candidate model terms:

(13) $$P=\left[p_{1}(t),p_{2}(t),\cdots ,p_{M}(t)\right]=\left[\begin{array}{llll} p_{1}(1) & p_{2}(1) & \cdots & p_{M}(1)\\ p_{1}(2) & p_{2}(2) & \cdots & p_{M}(2)\\ \vdots & \vdots & \ddots & \vdots \\ p_{1}(N) & p_{2}(N) & \cdots & p_{M}(N) \end{array}\right]$$

Although, from a physical structural perspective, the fatigue test bench discussed in this paper is a multi-input, multi-output (MIMO) system, this study models the system using four independent multi-input, single-output (MISO) NARX models, primarily based on the following engineering considerations: First, MIMO models have an extremely high parameter dimension, which can easily lead to redundancy and convergence difficulties in nonlinear system identification based on evolutionary algorithms; Second, the objective of this study is not to derive the global coupled dynamic equations for the entire working mechanism, but rather to use a specific critical failure point (such as CD_L1) as the damage assessment benchmark to retroactively estimate and reconstruct the fatigue load spectrum at that point. Although the various output points are physically coupled, in engineering applications, establishing a multi-input, single-output model specifically for a single critical point not only significantly reduces algorithmic complexity but also avoids inversion failure caused by the non-convexity of the Pareto front in multi-objective optimization, thereby more efficiently supporting the fatigue life assessment of structural weak points.

### 3.3. FROLS Parameter Identification Method

**Figure 2:**
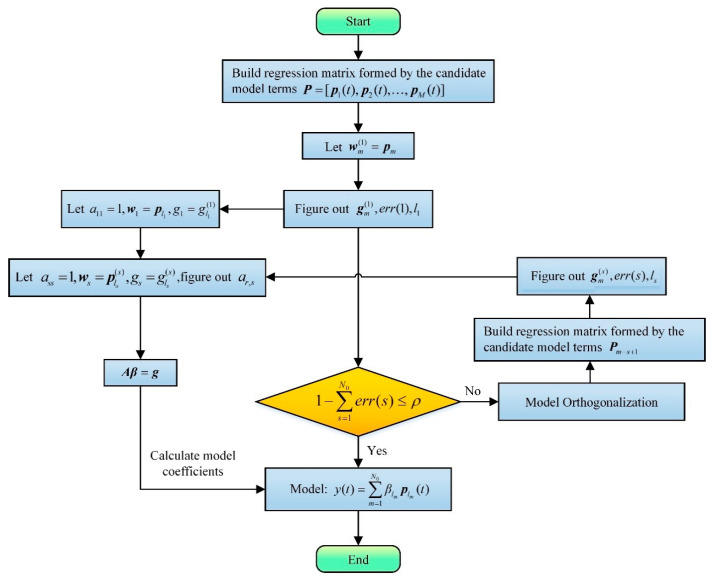
Modeling process of FROLS Algorithm.

For linear systems, the Least Squares (LS) algorithm can directly estimate model parameters. However, for nonlinear systems, the LS algorithm is no longer applicable. To address this issue, Billings and Korenberg [15,21] proposed the OLS algorithm and Error Reduction Ratio (ERR) criterion for nonlinear system identification. The basic idea of this method is to define and introduce an auxiliary model based on the orthogonal algorithm. Since all model terms in the auxiliary model are orthogonal to each other, the model coefficients can be estimated independently of other model terms. Finally, the actual system model can be obtained using the inverse orthogonal algorithm. As this algorithm has been increasingly applied and studied across various fields, it has been found that the ordering of model terms in the candidate matrix, particularly the position of pi(t) in Equation (6), may affect the identification results and even lead to incorrect model structures due to the characteristics of the ERR criterion. To address this issue, Billings et al. proposed the FROLS algorithm [22,23], building upon the OLS algorithm and ERR criterion. The FROLS algorithm incorporates a reordering process of model terms, in which the most significant term is selected from the remaining candidates at each step of the search procedure, ultimately yielding a parsimonious model structure. Figure 2 shows the modeling process of the FROLS algorithm. Due to the effective application of the FROLS algorithm in practical engineering problems, it has been widely used and remains one of the most effective algorithms for NARX model identification. The specific steps of the FROLS algorithm are as follows:

In the first step, disregarding the noise influence in Equation (12), let *w_m_*^(1)^ = *p_m_* and calculate:

(14) $$g_{m}^{\left(1\right)}=\frac{y^{T}w_{m}^{\left(1\right)}}{{\left(w_{m}^{\left(1\right)}\right)}^{T}w_{m}^{\left(1\right)}}$$

(15) $${ERR}^{\left(1\right)}[m]=\frac{{\left(g_{m}^{\left(1\right)}\right)}^{2}\left({\left(w_{m}^{\left(1\right)}\right)}^{T}w_{m}^{\left(1\right)}\right)}{y^{T}y}\times 100\%$$

(16) $$l_{1}=\arg\;\max\{ERR^{\left(1\right)}[m]\},\;1\leq m\leq M$$

In Equation (16), *argmax*{.} represents the model term as the l1 term when *ERR*^(1)^[*m*] takes its maximum value, which can be expressed as follows:

(17) $$eer(1)=ERR^{\left(1\right)}[l_{1}]$$

In step s(s ≥ 2), the column corresponding to $m\neq l_{1},m\neq l_{2},\ldots ,m\neq l_{s-1}$:

(18) $$w_{m}^{\left(s\right)}=p_{m}-\sum_{r=1}^{s-1}\frac{p_{m}^{T}w_{r}}{w_{r}^{T}w_{r}}w_{r}$$

(19) $$g_{m}^{\left(s\right)}=\frac{y^{T}w_{m}^{\left(s\right)}}{{\left(w_{m}^{\left(s\right)}\right)}^{T}w_{m}^{\left(s\right)}}$$

(20) $$ERR^{\left(s\right)}[m]=\frac{{\left(g_{m}^{\left(s\right)}\right)}^{2}\left[{\left(w_{m}^{\left(s\right)}\right)}^{T}\left(w_{m}^{\left(s\right)}\right)\right]}{y^{T}y}$$

(21) $$l_{s}=\arg\max\{ERR^{\left(s\right)}[m]\},\;1\leq m\leq M$$

During the execution of the algorithm, the algorithm terminates if the ESR satisfies the following conditions:

(22) $$ESR=1-\sum_{s=1}^{N_{0}}err(s)\leq \rho$$

Here, *N*_0_ represents the progress of the algorithm up to step *N*_0_; typically, *ρ* ≤ 0.01.

At this point, the model terms obtained through the FROLS algorithm can be described by the following expression:

(23) $$y(t)=\sum_{i=1}^{N_{0}}g_{i}w_{i}(t)$$

An alternative equivalent expression for the above equation can be written as:

(24) $$y(t)=\sum_{m=1}^{N_{0}}\beta_{l_{m}}p_{l_{m}}(t)$$

The coefficient vector $\beta ={\left[\beta_{l_{1}},\beta_{l_{2}},\ldots ,\beta_{l_{N0}}\right]}^{T}$ can be calculated using the following equation in the expression:

(25) Aβ=g

Among them,

(26) $$g={\left[g_{1},g_{2},\ldots ,g_{N_{0}}\right]}^{T}$$

(27) $$A=\left[\begin{array}{ccccc} 1 & a_{12} & a_{13} & \cdots & a_{1N_{0}}\\ 0 & 1 & a_{23} & \cdots & a_{2N_{0}}\\ \vdots & \vdots & 1 & \cdots & \vdots \\ 0 & 0 & \cdots & \ddots & a_{N_{0}-1,N_{0}}\\ 0 & 0 & 0 & \cdots & 1 \end{array}\right]$$

From this, we can obtain the numerical model that describes the nonlinear system, as shown by Equation (10).

It should be stated that the names and symbols of all alphabetic characters are listed in the Appendix A to facilitate understanding of the meanings of all variables.

## 4. Determining the Loading Spectrum Based on the NARX Model

Wheel loaders are one of the primary types of machinery frequently used in earthmoving operations. The operational methods vary significantly among users, and the working environments are generally harsh. In the design or improvement process of wheel loaders, if there is no durability test based on load spectrum data, substantial costs need to be invested in conducting tests at testing facilities or collecting data from users during actual usage. This approach not only involves various uncertainties but also has an extended time cycle. Therefore, it is critical to develop a method integrated with data-driven models to obtain load spectrum data for wheel loader working mechanisms, as this can drastically reduce intermediate costs throughout the entire process from design to practical application.

### 4.1. Algorithm-Based Load Identification Method

As a meta-heuristic optimization method that simulates the process of biological evolution, the basic idea behind genetic algorithms (GA) is to encode candidate solutions to a problem as “chromosomes.” Through three genetic operations—selection, crossover, and mutation—the population evolves generation by generation, allowing individuals with high fitness to be retained and reproduced, thereby gradually converging on the global optimal solution [24,25]. Compared with traditional gradient-based optimization methods, genetic algorithms do not rely on the continuity or differentiability of the objective function and exhibit good robustness for multi-peaked, nonlinear, and noisy problems; therefore, they demonstrate unique advantages in solving inverse problems such as structural parameter identification and load inversion. In this study, the genetic algorithm is employed as the core optimization tool to address the inverse load identification problem for the working mechanism of a wheel loader. The accuracy of fatigue test bench experiments depends to a large extent on the reliability of the load spectrum. However, due to structural and operational constraints, it is difficult to directly measure the load at the bucket cutting edge under actual working conditions. Consequently, a data-driven model [26,27] was established by combining the genetic algorithm with the NARX model; this model can determine the required input loads through a data-backward inference method. The Genetic Algorithm parameter configuration can be seen in the Appendix B.

**Figure 3:**
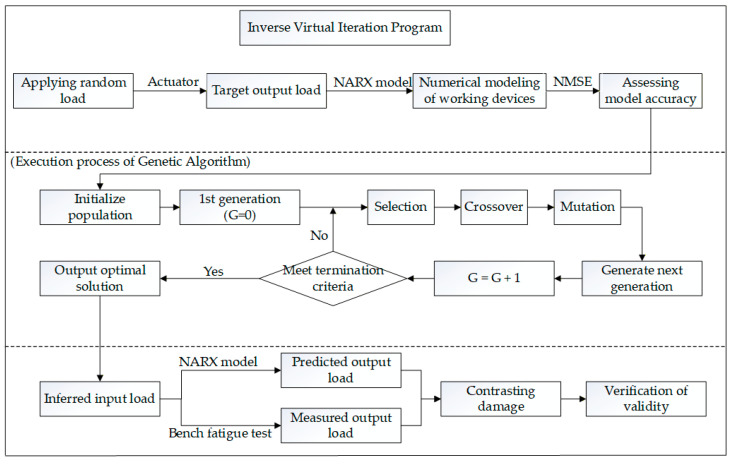
Technology roadmap.

First, random loads are applied to the four actuators of the wheel loader fatigue test bench, serving as system inputs and generating corresponding force and pressure responses at different locations of the working device. Dynamic loading data and the corresponding target output load spectra are collected through the test bench and strain gauges installed at various measurement points. Based on system identification techniques, a numerical model of the wheel loader working device is established using the NARX model to represent its dynamic characteristics. Subsequently, combining the established numerical model with the measured target output signals, a genetic algorithm is employed to perform iterative optimization, thereby reconstructing the input loads at the bucket cutting edge to reproduce the strain responses observed during actual operation. Finally, the identified input loads are applied to the fatigue test bench to generate corresponding output responses. These outputs are then compared with the target signals in terms of fatigue damage values, thereby validating the effectiveness and feasibility of the proposed approach. The overall workflow is summarized in Figure 3. It is worth noting that, theoretically, one could also utilize numerical models built on FEM or MBD to perform such reverse iterations. However, the working device of a wheel loader is an extremely large and complex multi-joint system. If FEM or MBD models were used as the fitness function for the genetic algorithm, the computational cost would be prohibitively high. In such optimization algorithms, it is necessary to repeatedly evaluate the system response for thousands of generations. The single forward calculation of a high-fidelity FEM/MBD model often takes minutes or even hours, making global optimization practically unfeasible. In contrast, the NARX model, once identified, acts purely as a set of algebraic equations (see Equation (34)). This enhances computational efficiency by orders of magnitude compared to traditional FEM/MBD simulations.

### 4.2. Numerical Example

**Figure 4:**
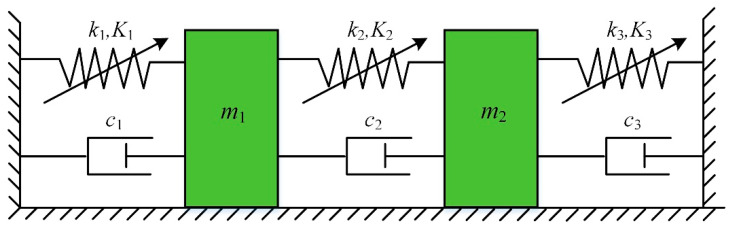
Two-degree-of-freedom damp vibration system.

In this section, we illustrate the correlation between the differential equations and the NARX model using a two-degree-of-freedom damping vibration system, as shown in Figure 4. Here, *m*_1_ and *m*_2_ represent the mass of the blocks, while *k*_1_, *k*_2_, and *k*_3_ represent linear stiffness, and *K*_1_, *K*_2_, and *K*_3_ represent nonlinear stiffness. The damping coefficients are represented by c_1_, c_2_, and c_3_. The system inputs are *u*_1_(*t*) and *u*_2_(*t*), and the outputs are *y*_1_(*t*) and *y*_2_(*t*). The differential equations governing the dynamics of this system are given by:

(28) $$\left\{\begin{array}{c} m_{1}{\ddot{y}}_{1}(t)+(c_{1}+c_{2}){\dot{y}}_{1}(t)-c_{2}{\dot{y}}_{2}(t)+(k_{1}+k_{2})y_{1}(t)+(K_{1}+K_{2})y_{1}^{3}(t)-k_{2}y_{2}(t)-K_{2}y_{2}^{3}(t)=u_{1}(t)\\ m_{2}{\ddot{y}}_{2}(t)-c_{2}{\dot{y}}_{1}(t)+(c_{2}+c_{3}){\dot{y}}_{2}(t)-k_{2}y_{1}(t)+(k_{2}+k_{3})y_{2}(t)-K_{2}y_{1}^{3}(t)+(K_{2}+K_{3})y_{2}^{3}(t)=u_{2}(t) \end{array}\right.$$

In the equations, *u*_1_(*t*) and *u*_2_(*t*) correspond to the system inputs at time *t*. $y_{1},{\dot{y}}_{1},{\ddot{y}}_{1}$ represent the displacement, velocity, and acceleration of mass block *m*_1_, while $y_{2},{\dot{y}}_{2},\;{\ddot{y}}_{2}$ signify the displacement, velocity, and acceleration of mass block *m*_2_.

Assuming the displacement of mass block *m*_2_ is zero, the differential equations governing the dynamics of this system are as follows:

(29) $$m_{1}{\ddot{y}}_{1}(t)+c_{1}{\dot{y}}_{1}(t)+k_{1}y_{1}(t)+K_{1}y_{1}^{3}(t)=u_{1}(t)+u_{2}(t)$$

Equation (29) represents the Duffing equation, widely employed in engineering to describe the dynamic behavior of nonlinear systems. Based on the study of nonlinear problems using the Duffing equation described in References [28,29]. Thus, in this section, we use this dynamic equation as a general illustration. The first-order derivatives and second-order derivatives in Equation (29) can be approximated as follows:

(30) $${\dot{y}}_{1}(t)=\frac{y_{1}(t)-y_{1}(t-1)}{\Delta t}$$

(31) $${\ddot{y}}_{1}(t)=\frac{y_{1}(t+1)-2y_{1}(t)+y_{1}(t-1)}{\Delta t^{2}}$$

where *t* represents the sampling points, and Δt denotes the sampling interval.

The following discrete expressions are obtained by substituting Equations (30) and (31) into Equation (29):

(32) $$\begin{array}{l} y_{1}(t)=\frac{\Delta t^{2}}{m_{1}}u_{1}(t-1)+\frac{\Delta t^{2}}{m_{1}}u_{2}(t-1)+\frac{2m_{1}-c_{1}\Delta t-k_{1}\Delta t^{2}}{m_{1}}y_{1}(t-1)\\ \;\;\;\;-\frac{m_{1}-c_{1}\Delta t}{m_{1}}y_{1}(t-2)-\frac{K_{1}\Delta t^{2}}{m_{1}}y_{1}^{3}(t-1) \end{array}$$

Based on Equations (22) and (24), it can be observed that the discrete form of the Duffing equation, as presented in Equation (32), shares a resemblance with the NARX model structure. This preliminary observation suggests a potential correlation between the two. To further elucidate the relationship between Equation (32) and the NARX model, we define the parameters of the two-degree-of-freedom damped vibration system shown in Figure 4 as follows: *m*_1_ = 15 kg; *k*_1_ = 3.56 × 105 Nm^−1^; *K*_1_ = 6.85 × 107 Nm^−3^; *c*_1_ = 600 Nms^−1^. Utilizing these parameters, the discrete model describing the dynamic system depicted in Figure 4 can be obtained as follows:

(33) $$y_{1}(t)=2.54\times 10^{-7}u_{1}(t-1)+2.54\times 10^{-7}u_{2}(t-1)+1.83y_{1}(t-1)-0.92y_{1}(t-2)-17.42y_{1}^{3}(t-1)$$

**Figure 5:**
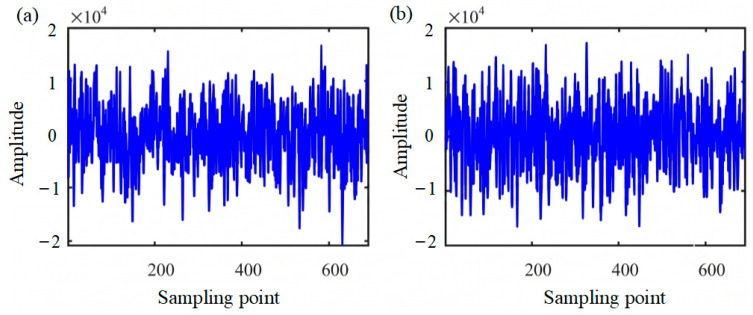
The random input loads applied to the two-degree-of-freedom damped vibration system: (**a**) input load *u*_1_(*t*); (**b**) input load *u*_2_(*t*).

To verify the correlation between the numerical model obtained using the FROLS algorithm and the differential equation, we define the input signal *u_i_* (t) as a random signal following a uniform distribution, where the signals are shown in Figure 5. Next, we use the Runge–Kutta method to numerically solve the differential equation given in Equation (29) with a sampling frequency of *f_s_* = 512 Hz. The Runge–Kutta method provides a highly accurate and stable numerical integration scheme for ordinary differential equations and is one of the most widely used tools for engineering dynamics simulations [29,30]. Based on the random input signal and the corresponding output signal, we configure the system identification parameters as follows: the maximum input delay *n_u_* = 2, the maximum output delay *n_y_* = 2, and the highest order *l* = 3. Employing the FROLS algorithm, we derive the numerical model that describes the two-degree-of-freedom damped vibration system shown in Figure 4 as follows:

(34) $$\begin{array}{l} y_{1}(t)=1.8262y_{1}(t-1)-0.9239y_{1}(t-2)+1.2357\times 10^{-7}u_{2}(t-1)+1.2368\times 10^{-7}u_{1}(t-1)\\ \;\;\;\;\;+1.2073\times 10^{-7}u_{1}(t-2)+1.2087\times 10^{-7}u_{2}(t-2) \end{array}$$

**Figure 6:**
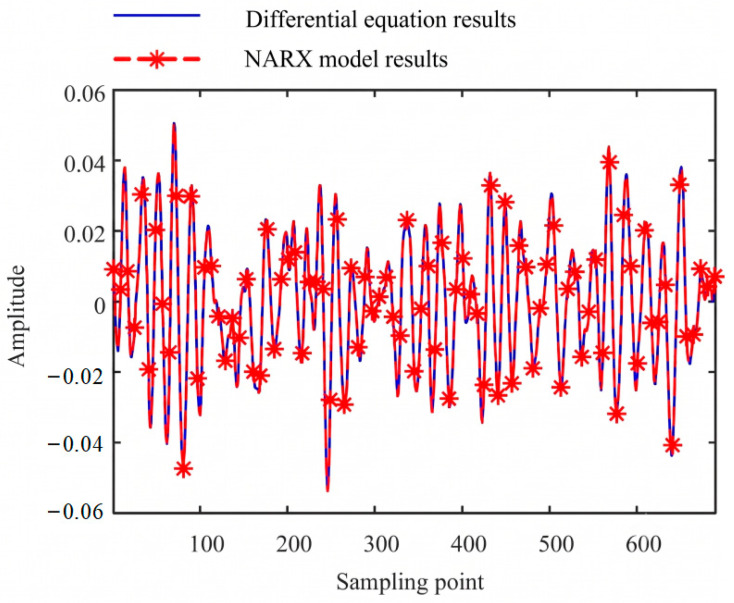
Comparison between NARX model output and results obtained by solving the differential equation.

Equations (33) and (34), it can be observed that the NARX model obtained using the FROLS algorithm is similar to the discrete form given in Equation (29), with slight differences in the model terms. To further illustrate the distinction between the two, the identification results and the model structure shown in Equation (33) are listed in Table 1 (arranged in descending order based on the ERR values). The identified model terms include all the real terms, and the coefficients of the corresponding model terms are generally consistent. However, the identification results reveal two additional model terms, respectively *u*_1_(*t* − 2) and *u*_2_(*t* − 2), highlighted in bold in Table 1. Furthermore, it can be observed that the sum of the coefficients of *u*_1_(*t* − 1) and *u*_1_(*t* − 2) is close to the coefficient of *u*_1_(*t* − 1) in the discrete expression, and similarly, the sum of the coefficients of *u*_2_(*t* − 1) and *u*_2_(*t* − 2) is close to the coefficient of *u*_2_(*t* − 1) in the discrete expression. This is due to the fact that the sum of the model terms *u_i_*(*t* − 1) and *u_i_*(*t* − 2) is close to the real term *u_i_*(*t* − 1), and the numerical model obtained using the FROLS algorithm can approximately represent the true model. This phenomenon is extremely common in the identification of nonlinear systems based on finite sampling frequencies. Since the sampling interval Δt in this example is relatively small, during the discretization process, the system’s single input delay term u(t − 1) is jointly represented by the adjacent terms u(t − 1) and u(t − 2), thereby generating multicollinearity and redundant terms. This is a typical example of term structure dispersion in the FROLS algorithm caused by frequency response characteristics. Such phenomena are common in NARX model identification based on the FROLS algorithm. Therefore, for such engineering nonlinear systems, we should not regard the NARX model as a strict physical equation equivalent, but rather as an input–output equivalent surrogate model. The most important metric for evaluating the quality of such surrogate models is not whether the model terms are completely consistent with the differential equations, but whether the error between the model-predicted output (MPO) and the actual system response meets the requirements of engineering applications. To demonstrate the accuracy of the numerical model, the system response is obtained based on the MPO model verification method and compared with the results obtained by solving the differential equation using the Runge–Kutta method, as shown in Figure 6. The system response obtained based on the numerical model aligns well with the results obtained from solving the differential equation. The above analysis demonstrates the feasibility of using the numerical model to represent the dynamic system in place of the differential equation. 

It should be noted that the above process is often used as a validation method for the accuracy of numerical modeling results in simple dynamical systems. For complex nonlinear systems, especially those with a large number of degrees of freedom, obtaining the model structure as shown in Equation (33) is difficult. In such cases, the model-predicted outputs can be compared with the measured results to validate the accuracy of the numerical model.

**Figure 7:**
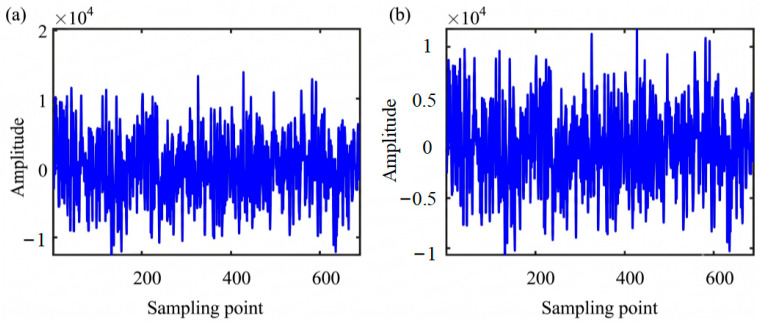
Input signals reconstructed by the genetic algorithm for the two-degree-of-freedom damped vibration system: (**a**) inverted signal *u*_1_(*t*); (**b**) inverted signal *u*_2_(*t*).

**Figure 8:**
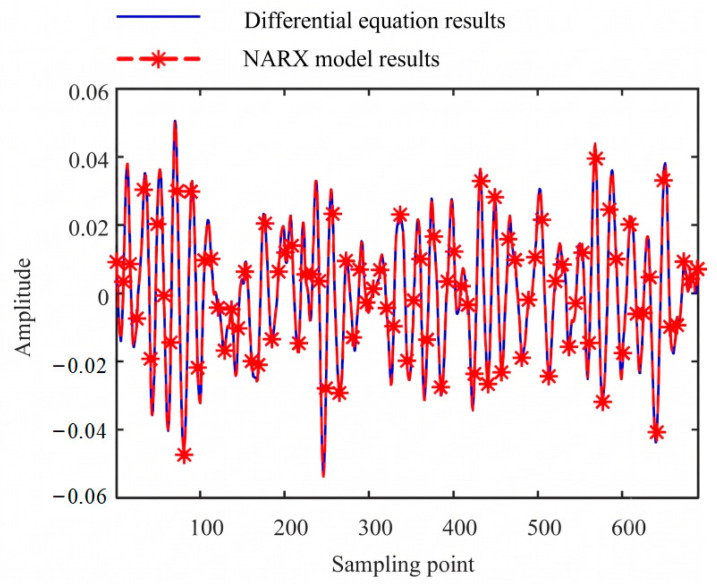
Comparison of the output predicted by the inverted input signal through the NARX model with the results of the differential equation solution.

The established NARX model is used as the fitness function, while a genetic algorithm is employed to perform inverse virtual iteration for input signal reconstruction. The reconstructed input is then applied to the NARX model to predict the output response, where the input signals are given in Figure 7. The error between the predicted and theoretically calculated outputs is subsequently evaluated. The results are shown in Figure 8.

The output obtained from the input signal reconstructed by the genetic algorithm matches well with the measured response, indicating that the NARX-based numerical model can accurately capture the system’s dynamic characteristics. 

## 5. Experimental Applications

**Figure 9:**
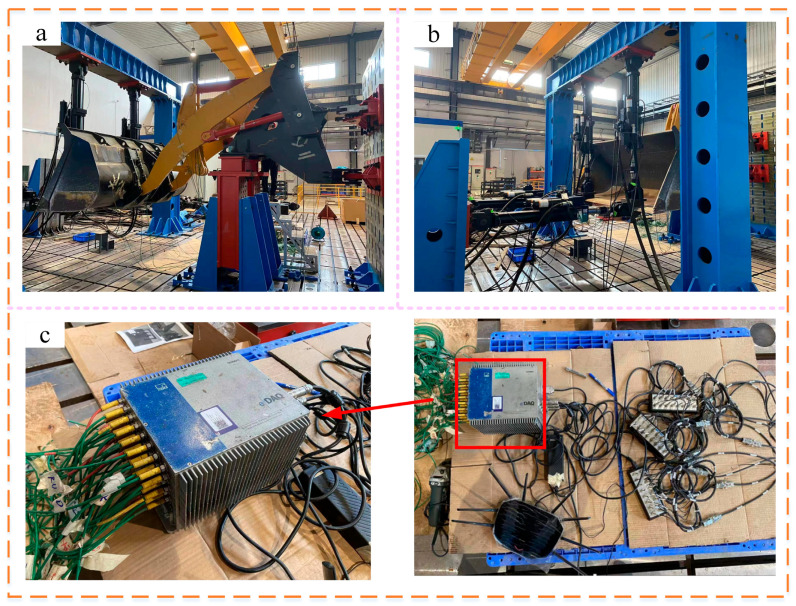
(**a**) Loader working device; (**b**) actuators of the fatigue testing platform; (**c**) signal acquisition device.

The numerical example conducted earlier for inferring input loads using the inverse VIM was relatively simple and did not include experimental verification to demonstrate its effectiveness and applicability. To validate the proposed method under realistic conditions, the following experiment was conducted on a wheel loader working device mounted on a fatigue test rig. As shown in Figure 9a, the wheel loader’s working device is mounted on the fatigue test rig, and four actuators apply random excitation signals from 0 to 7 Hz to the bucket tip of the wheel loader’s working device (see Figure 9b). The output response signals of each measurement point were collected by a data acquisition device (see Figure 9c).

### 5.1. The Modeling of the Wheel Loader’s Working Mechanism Based on a Multiple-Input Single-Output NARX Model

Prior to performing system identification with the FROLS algorithm, the structural parameters of the NARX model—specifically the input time delay $n_{u}$, output time delay $n_{y}$, and the highest nonlinear order l—must be determined rationally. Specifically, the search ranges were set to $n_{u},n_{y}\in [1,4]$ and l∈[1,4]. For each combination of (*n_u_*, *n_y_*, *l*), the system was identified using the FROLS algorithm, and Normalized Mean Square Error (NMSE) was used as the core evaluation metric for model accuracy. Therefore, considering the trade-off among fitting accuracy, model complexity, and computational efficiency, the final system identification parameters are selected as $n_{u}=2,n_{y}=2,$ and l=3. The FROLS algorithm is utilized for system identification to establish a multi-input single-output NARX model for the working device.

**Figure 10:**
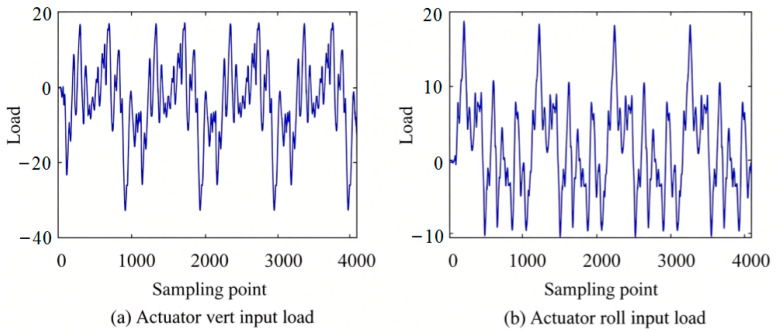

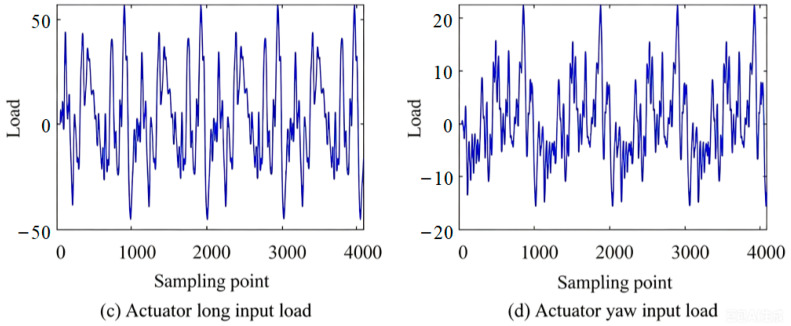
Random input load in the range of 0 to 7 Hz.

**Figure 11:**
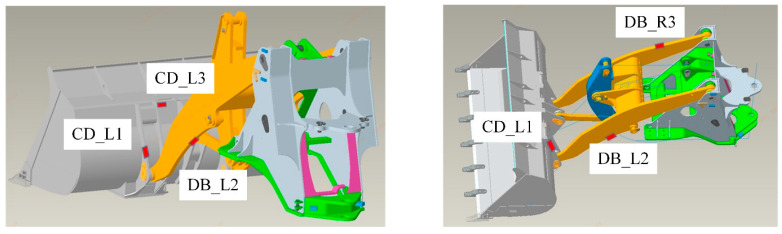
Description of measurement points.

To better adapt to the variations in the working device during actual operation and enhance the accuracy and predictive capability of the model, we define the input signals *u*_1_(*t*), *u*_2_(*t*), *u*_3_(*t*) and *u*_4_(*t*) as the random load signals (ranging from 0 to 7 Hz) applied to the four actuators of the test bench (vert, roll, long and yaw). The output signal is the micro-strain measured by strain gauges mounted on the working device of the wheel loader, with a sampling frequency of *f* = 200 Hz, where the random input load are shown in Figure 10 and the measurement point can be seen in Figure 11.

The numerical model for measurement point CD_L1 is as follows:

(35) $$\begin{array}{l} y(t)=\;1.7978y(t-1)-0.8105y(t-2)+0.2879u_{4}(t-2)-0.3017u_{4}(t-1)-\\ \;\;\;\;\;0.4387u_{1}(t-1)+0.3642u_{1}(t-2)-0.1564u_{3}(t-1)+0.1294u_{3}(t-2)+\\ \;\;\;\;\;8.7724\times 10^{-4}u_{2}(t-2)\times u_{1}(t-2)+2.0832\times 10^{-5}u_{3}(t-2)\times u_{4}{\left(t-2\right)}^{2}-\;\\ \;\;\;\;\;0.2614\times 10^{-4}u_{2}(t-1)+0.2447\times \;10^{-4}u_{2}(t-2)+1.2995\times 10^{-6}u_{2}(t-1)\times y{\left(t-1\right)}^{2} \end{array}$$

The numerical model for measurement point DB_L2 is as follows:

(36) $$\begin{array}{l} y(t)=\;1.8787y(t-1)-0.8933y(t-2)+0.1775u_{4}(t-2)-0.0744u_{1}(t-1)-0.1994u_{4}(t-1)\\ \;\;\;\;\;+5.0732\times 10^{-4}u_{3}{\left(t-1\right)}^{3}-0.0835u_{3}{\left(t-2\right)}^{2}+6.6726\times 10^{-5}u_{2}(t-2)\times u_{4}(t-2)+\\ \;\;\;\;\;0.0509u_{2}(t-2)\times u_{4}(t-1)-0.1169u_{3}(t-2)\times u_{4}{\left(t-1\right)}^{2}+0.0947u_{3}(t-1)\\ \;\;\;\;\;-5.04\times 10^{-4}u_{3}(t-1)\times u_{4}(t-1)\times u_{4}(t-2)+4.5661\times 10^{-4}u_{2}(t-1) \end{array}$$

The numerical model for measurement point CD_L3 is as follows:

(37) $$\begin{array}{l} y(t)=\;1.9611y(t-1)-1.1355y(t-2)+0.1604y(t-3)-0.5939u_{1}(t-1)+0.4947u_{1}(t-2)\\ \;\;\;\;\;+0.2100u_{4}(t-2)-0.2230u_{4}(t-1)-0.1978u_{3}(t-1)+0.1637u_{3}(t-2)-0.3811u_{2}(t-1)\\ \;\;\;\;\;+0.3526u_{2}(t-2)-4.4632\times 10^{-4}u_{1}(t-1)\times y(t-3)+4.0514\times 10^{-4}u_{1}(t-2)\times y(t-1) \end{array}$$

The numerical model for measurement point DB_R3 is as follows:

(38) $$\begin{array}{l} y(t)=\;1.8542y(t-1)-0.8668y(t-2)+0.2178u_{1}(t-1)-0.1567u_{1}(t-2)\\ \;\;\;\;\;+0.0759u_{3}(t-1)+0.0547u_{2}(t-1)-0.0515u_{3}(t-2)-0.0413u_{2}(t-2)\\ \;\;\;\;\;-4.6233\times 10^{-5}u_{1}(t-1)\times y(t-2)+0.0044u_{4}(t-1)-3.3701\times 10^{-4}u_{3}{\left(t-2\right)}^{2}\\ \;\;\;\;\;+3.2354\times 10^{-4}u_{3}{\left(t-1\right)}^{2}+4.1185\times 10^{-5}u_{4}(t-2)\times y(t-2) \end{array}$$

**Figure 12:**
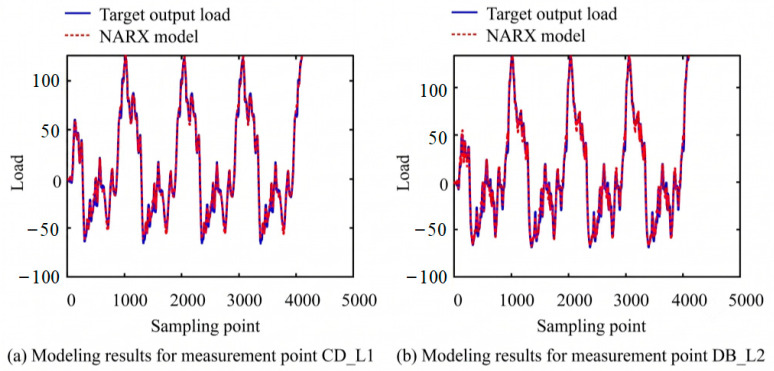

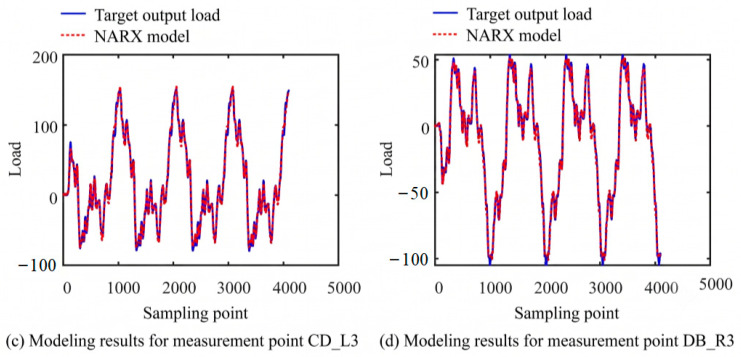
Comparison chart of NARX numerical model output results versus target output loads at different measurement points on the wheel loader’s working mechanism.

During this process, the FROLS algorithm iteratively seeks the optimal model terms by analyzing and processing both input and output signals. It aims to make the model output as close as possible to the measured output. Equation (35) to (38) illustrates the NARX numerical model of the working mechanism of the wheel loader, established using the FROLS algorithm. It compares the model output signal for a given input with the target output. This comparison allows for the evaluation of the fitting degree and accuracy of the NARX model. From Figure 12 it is evident that there is a high level of conformity between the NARX numerical model and the target output. This indicates that the numerical model of the loader, established using the NARX approach, can accurately predict the actual output load. Through identification using the FROLS algorithm, the NARX model successfully captures the key features and behaviors of the real system. By incorporating lag and nonlinear terms, the NARX model effectively describes the dynamic characteristics of the system. To further assess the difference between the NARX numerical model and the actual output, NMSE is used to evaluate model accuracy.

As shown in Table 2, the multi-input single-output NARX model accurately captures the dynamic behavior of the working device of the wheel loader. Throughout the process, the FROLS algorithm meticulously analyzes and processes the input and output signals, iteratively searching for the optimal model terms to ensure a close match between predicted outputs and actual measured target outputs. As shown in Table 2, the multi-input single-output NARX model accurately captures the dynamic behavior of the working device of the wheel loader. As a result, a reliable numerical model is established, effectively reflecting the system dynamic characteristics consistent with real-world observations. In this context, the closer R^2^ is to 1, the better the model fits the data.

**Figure 13:**
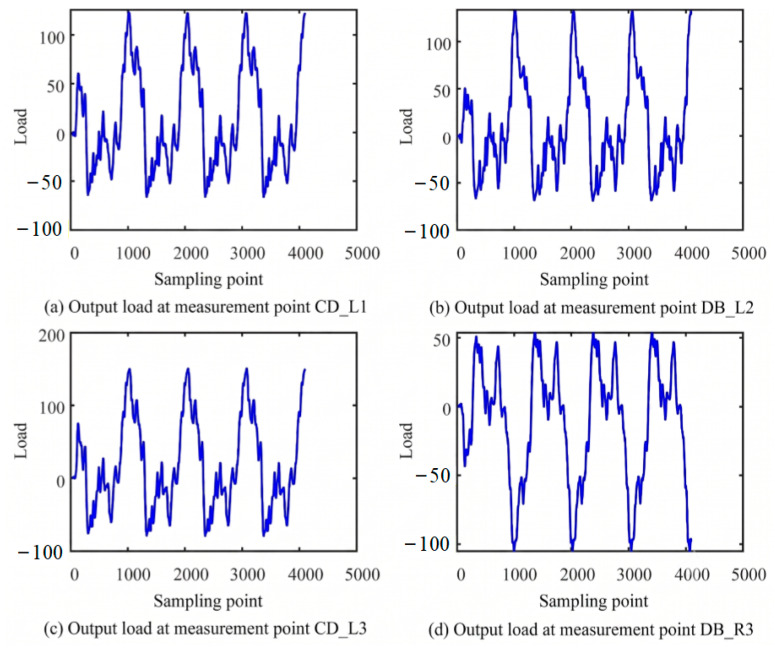
Output load at each measurement point.

It is worth noting that, as shown in Figure 13c, the output load amplitude at measurement point CD_L3 (ranging from −50 to +150) is significantly higher than at the other three measurement points. Consequently, the denominator of the NMSE at CD_L3 (i.e., the variance of the target signal) is significantly larger. At the same time, the MSE value at CD_L3 remains extremely small, indicating that the NARX model exhibits exceptional accuracy in capturing the output response under these high-amplitude conditions. Consequently, although the numerator (MSE) remains consistently minimal and stable, the increase in the denominator causes the NMSE value at CD_L3 to appear approximately two orders of magnitude lower than at other measurement points. This phenomenon does not stem from overfitting or anomalies in the training/test set split, but rather reflects the inherent statistical characteristics of the NMSE metric when evaluating measurement points with large variations in load amplitude, and is therefore a normal occurrence.

### 5.2. Load Spectrum Back Calculation Based on Multi-Input Single-Output NARX Model

**Figure 14:**
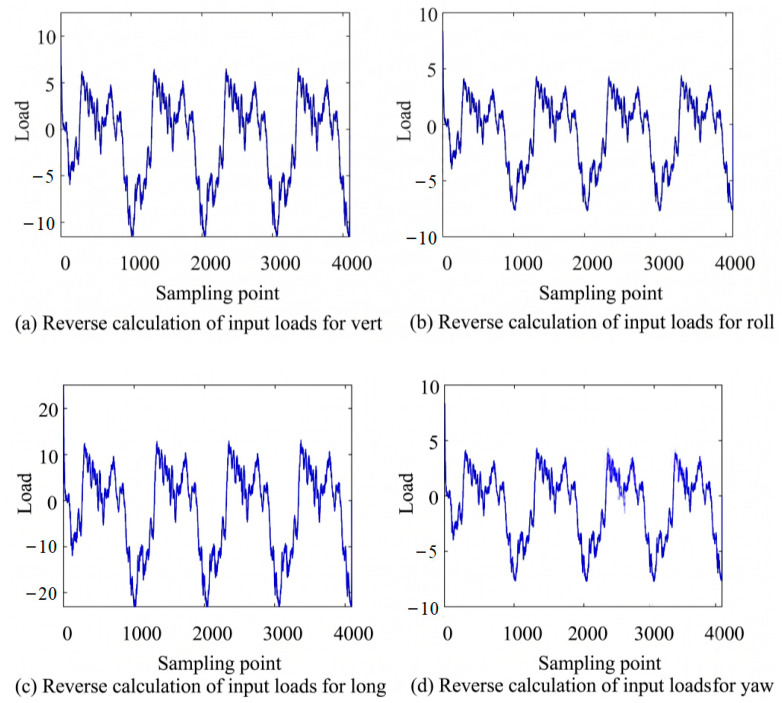
Derived input loads for the four actuators of the fatigue test platform determined using a genetic algorithm.

**Figure 15:**
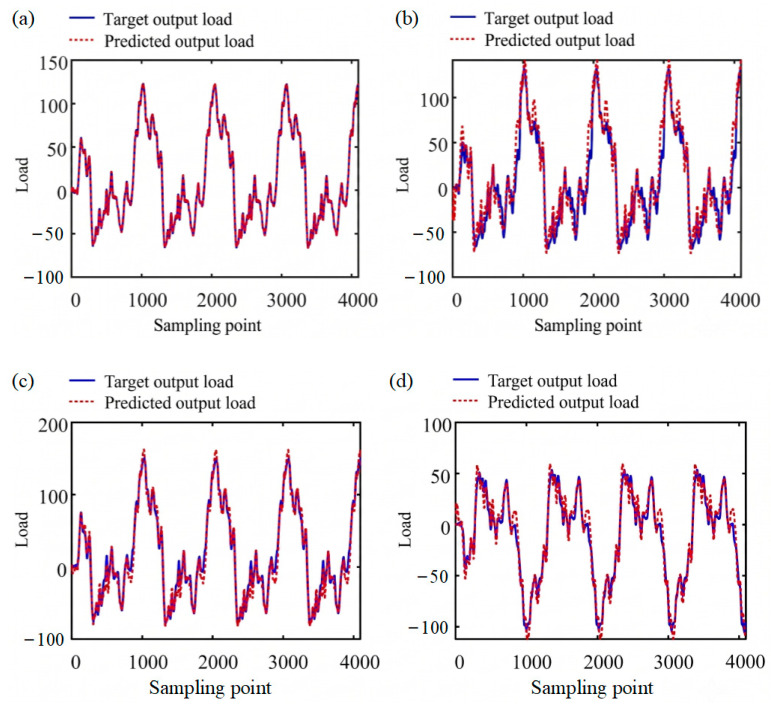
Comparison of the output load predicted by the NARX model with the target output load at four measurement points: (**a**) Measurement point CD_L1; (**b**) Measurement point DB_L2; (**c**) Measurement point CD_L3; (**d**) Measurement point DB_R3.

Using the NARX numerical model at measurement point CD_L1 as the fitness function for the genetic algorithm, the inputs loads for the four actuators were inversely inferred through inverse virtual iteration, as shown in Figure 14. This inferred load was then utilized as excitation. Through the established NARX numerical model, the output loads at measurement points were predicted and compared with the target output loads. The results are as follows:

From Figure 15, it can be observed that the predicted output loads at measurement point CD_L1 fit well with the target output loads, while there are slight discrepancies at other measurement points. This is because the genetic algorithm requires a fitness function to represent the problem being solved. The numerical model of the working device established based on the NARX model cannot be completely error-free. Therefore, when the NARX model at a specific measurement point is used as the fitness function for the genetic algorithm, good agreement is achieved at that point, resulting in discrepancies between the predicted and target outputs at other measurement points. However, the overall error remains small, indicating that the NARX-based numerical model of the working device of the wheel loader can accurately capture the key characteristics of the real system. It demonstrates consistent dynamic characteristics with the actual situation. 

### 5.3. Experimental Results Analysis

**Figure 16:**
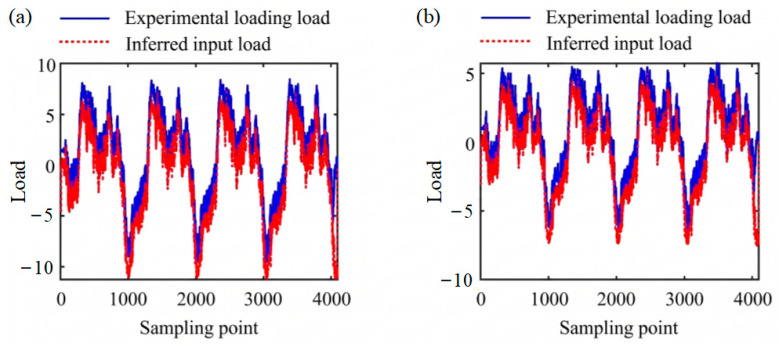

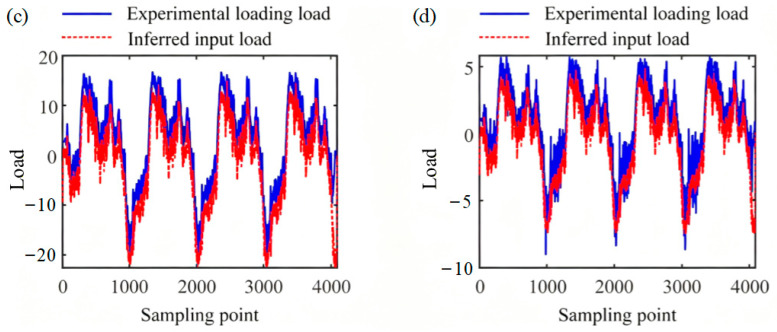
Comparison of experimentally loaded approximated loads with inferred input loads: (**a**) Actuator vert; (**b**) Actuator roll; (**c**) Actuator long; (**d**) Actuator yaw.

**Figure 17:**
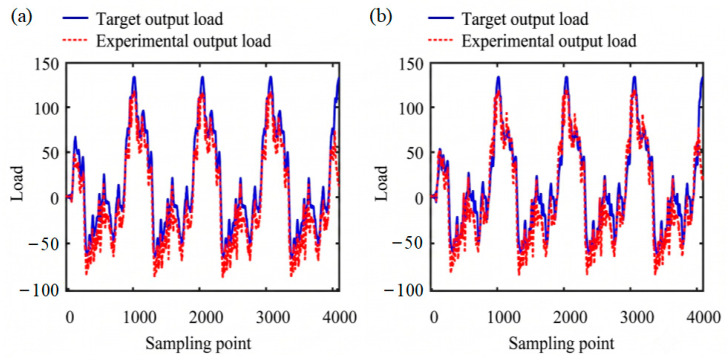

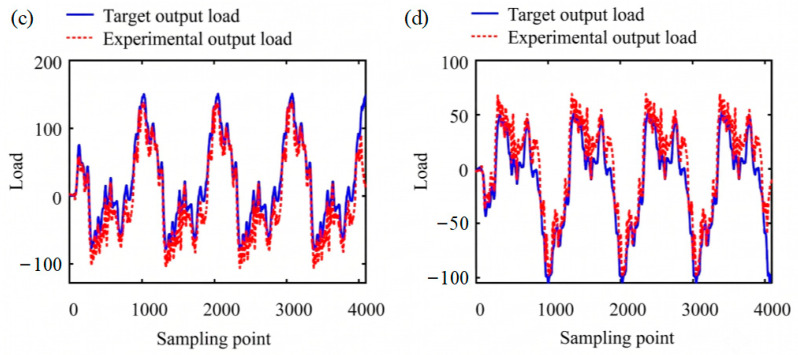
Comparison of experimental output load and target output load at each measurement point: (**a**) Measurement point CD_L1; (**b**) Measurement point DB_L2; (**c**) Measurement point CD_L3; (**d**) Measurement point DB_R3.

In this experiment, the aforementioned input loads were applied to the four actuators of the fatigue test rig. It is important to note that during the fatigue test on the test rig, due to limitations of the experimental equipment, the inferred input loads were unable to be directly applied to the actuators due to their high frequency. Therefore, an approximate load approximating the inferred input loads was used instead, this load mitigates the effects of high-frequency oscillations while preserving the primary cyclic amplitude sequence and the overall load trend. Figure 16 shows a comparison between the approximate load applied in the experiment and the identified input load. As can be seen from the figure, the approximate load accurately reflects the overall trend and general characteristics of the identified input load; its amplitude and frequency are essentially consistent with those of the actual input load, which meets our requirements for the approximate load. This is because the overall objective of this experiment is not to achieve perfect waveform matching, but rather to reproduce the cumulative fatigue damage caused by the dominant load cycle. The approximate load was then applied to the four actuators of the test rig, and the output loads at various measurement points of the working device were measured using a signal acquisition device, as shown in Figure 17.

As shown in Figure 17, a comparison of the experimental output with the target output at different measurement points reveals excellent results, with no significant differences observed. This indirectly demonstrates that this approximate load can replace the actual input load to reflect the cumulative fatigue damage it causes. It verifies the effectiveness and feasibility of the proposed method. Of course, a small number of cases exhibit minor deviations, these deviations are primarily attributable to the physical limitations of the test bench in reproducing high-frequency impact components and do not indicate any flaws in the reverse virtual iteration method. To further evaluate the engineering implications of these waveform deviations, fatigue damage calculations were performed using a virtual S-N curve, and the results are shown in Table 3. As shown in Table 3, despite noticeable differences in waveform details, the cumulative fatigue damage generated by the approximate loads exhibits excellent consistency with the target damage output results. The maximum error (ERR) at measurement point CD_L3 is approximately 14.41%, while the deviations at other measurement points are significantly lower. In the fatigue design of heavy-duty construction machinery—where loads are inherently random and unstable—damage deviations within this range (typically <15–20%) are considered entirely acceptable and, in practice, sufficient to guide structural design and durability verification. Despite variations in the waveforms, the consistency of the cumulative damage confirms that the proposed method successfully captures the influence of operational loads on fatigue.

From Figure 16 and Table 3, it is evident that the approximate load applied in the experiment captures the overall trend of the inferred input loads, accurately reproducing the strain experienced during operation. This indicates that the method of inverse virtual iteration to deduce input loads based on the NARX model and genetic algorithm holds certain reference value for determining the loading spectrum of working devices in engineering machinery. 

## 6. Conclusions

This study addresses the difficulty of directly obtaining input load spectra for construction machinery. To solve this problem, an inverse VIM combining a NARX model and a genetic algorithm is proposed and applied to a wheel loader’s working device. First, random loads are applied to the fatigue test bench, and the corresponding output load spectra are measured. A NARX-based numerical model of the working device is then established through data identification. Based on this model, a genetic algorithm is used to estimate the input load spectra. The calculated inputs are applied to the test bench, and the resulting outputs are compared with the target outputs. The effectiveness and feasibility of the method are validated through damage assessment. The main conclusions are as follows:

1. The study explains the multi-input single-output NARX model and the FROLS parameter identification method. Using the FROLS algorithm to perform iterative analysis of input–output data to determine the optimal model terms, a high-precision numerical surrogate model of a wheel loader’s working mechanism was successfully established. Compared to traditional methods, this data-driven modeling approach can significantly improve the computational efficiency of inverse iterative calculations for large-scale structures and reduce costs, making it a highly practical technical tool for fatigue testing in construction machinery.
2. Based on the established model, a genetic algorithm is iteratively optimized to infer the input loads for the four actuators of the fatigue test bench. The predicted output load spectra show high agreement with the target spectra, demonstrating the effectiveness and feasibility of the method.
3. Constrained by the dynamic response characteristics of the test equipment, the test bench cannot load the subtle high-frequency components within the input load spectrum. Accordingly, this study adopts approximate test loads with similar fatigue cycle characteristics to replace the real input loads for bench testing. A series of experiments and corresponding results verify the feasibility of the proposed method, which delivers a stable, reliable and directly industrially applicable technical scheme for compiling fatigue load spectra of heavy-duty construction machinery.

## Figures and Tables

**Table 1:** Identification results of the two-degree-of-freedom damped vibration system shown in Figure 4.

Step	Frequency Sweep System Identification Approach			Real System	
	Term	Coefficient		Term	Coefficient
1	*y*_1_(*t* − 1)	1.8262	90.22	*y*_1_(*t* − 1)	1.83
2	*y*_1_(*t* − 2)	−0.9239	8.96	*y*_1_(*t* − 2)	−0.92
3	*u*_1_(*t* − 1)	1.2368 × 10^−7^	0.23	*u*_1_(*t* − 1)	2.54 × 10^−7^
4	*u*_1_(*t* − 2)	1.2073 × 10^−7^	0.19	*u*_2_(*t* − 1)	2.54 × 10^−7^
5	*u*_2_(*t* − 1)	1.2357 × 10^−7^	0.19	*y*_1_ (*t* − 1)^3^	−17.42
6	*u*_2_(*t* − 2)	1.2087 × 10^−7^	0.20		
Total			99.99		

**Table 2:** Normalized mean square error for different measurement points in the NARX model in Figure 12.

Position	CD_L1	DB_L2	CD_L3	DB_R3
NMSE	2.4701 × 10^−4^	1.6351 × 10^−4^	1.2483 × 10^−6^	3.9320 × 10^−4^
R^2^	0.999753	0.999836	0.999999	0.999607

**Table 3:** Comparison of damage outputs at each measurement point.

Position	Target Output Load	Experimental Measured Output Load	ERR%
CD_L1	2.83857 × 10^−5^	2.90100 × 10^−5^	2.199634
DB_L2	3.45432 × 10^−5^	3.78371 × 10^−5^	9.535641
CD_L3	5.10631 × 10^−5^	5.84219 × 10^−5^	14.411127
DB_R3	1.65430 × 10^−5^	1.78262 × 10^−5^	7.756807

## Data Availability

The original contributions presented in this study are included in the article. Further inquiries can be directed to the corresponding author.

## Abbreviations

The following abbreviations are used in this manuscript:

ARX	Auto-Regressive with Extra inputs
CAE	computer-aided engineering
ERR	Error Reduction Ratio
FEM	Finite Element Model
FROLS	Forward Regression Orthogonal Least Squares
MDB	Multi-Body Dynamics
MPO	Model predicted output
NARX	Nonlinear Auto-Regressive with exogenous
NMSE	Normalized Mean Square Error
OLS	Orthogonal Least Squares
VIM	Virtual Iteration Method

## Appendix A

**Table A1:** The names and operators of all letters in the text.

Nomenclature			
$c_{i}$	Damping coefficients	t	*tth* data point in the data set
d	Time delay	u(t)	Input signals
e(k)	Independent signal relative to the input–output signals	y	System output matrix
$k_{i}$	Linear stiffness	y(t)	Output signals
$K_{i}$	Nonlinear stiffness	$y_{\mathrm{real}}(t)$	Actual output at time instant *t*
l	Degree of the model	${\dot{y}}_{1}$	Velocity of mass block *m*_1_
M	The number of total candidate model terms	${\dot{\ddot{y}}}_{1}$	Acceleration of mass block *m*_1_
$n_{u}$	Maximum time delays for the input and output sequences	${\dot{y}}_{2}$	Velocity of mass block *m*_2_
$n_{y}$	Maximum time delays for the input and output sequences	${\ddot{y}}_{2}$	Acceleration of mass block *m*_2_
$N_{0}$	Algorithm’s progress	$\theta_{i}$	Coefficients
P	Regression matrix formed by the candidate model terms	Operator	
$p_{i}(t)$	Model terms	*F*[.]	Unknown function to be identified

## Appendix B

### Genetic Algorithm Parameter Configuration

To ensure the reproducibility and transparency of the proposed inverse virtual iteration method, the specific configuration of the genetic algorithm (GA) implemented in MATLAB (R2024b) is explicitly detailed herein. GA employs a real-valued encoding scheme that maps binary chromosomes to continuous load variables via the bs2rv function. The core initialization parameters, derived directly from the experimental code implementation, are set as follows: the crossover probability is pc = 0.7, the mutation probability is pm = 0.05, the population size is NIND = 10, the maximum number of generations is MAXGEN = 10, the precision of each binary variable is PRECI = 10, the number of variables is NVAR = 3, and the generation gap is GGAP = 0.9. To confine the optimization within a feasible search space, the regional descriptors (FieldD) are defined using the precision and variable bounds (e.g., FieldD1 = [repmat (PRECI, [1, NVAR]); [−1, −1, −1; 1, 1, 1] *M1; repmat ([1; 0; 1; 1], [1, NVAR])]; where M1 represents the upper bound of the optimization variable amplitudes). The GA employs a roulette wheel selection operator, a multi-point crossover operator (xovmp), and a discrete mutation operator (mut) to maintain population diversity. Furthermore, an elite preservation strategy is implemented via fitness-based reinsertion (reins) with a generation gap of GGAP = 0.9. The evolutionary process terminates when the algorithm reaches the maximum generation of MAXGEN = 10, or when the change in optimal fitness is less than 10^−6^ for 20 consecutive generations.
